# Open pre-schools at integrated health services—A program theory

**DOI:** 10.5334/ijic.983

**Published:** 2013-04-12

**Authors:** Agneta Abrahamsson, Kerstin Samarasinghe

**Affiliations:** University College of Kristianstad, Sweden and Jönköping Academy, University of Jönköping, Sweden; University College of Kristianstad, Sweden

**Keywords:** integrated family centres, family support, program theory, parent empowerment, child health, multi-site design, professional compliance

## Abstract

**Introduction:**

Family centres in Sweden are integrated services that reach all prospective parents and parents with children up to their sixth year, because of the co-location of the health service with the social service and the open pre-school. The personnel on the multi-professional site work together to meet the needs of the target group. The article explores a program theory focused on the open pre-schools at family centres.

**Method:**

A multi-case design is used and the sample consists of open pre-schools at six family centres. The hypothesis is based on previous research and evaluation data. It guides the data collection which is collected and analysed stepwise. Both parents and personnel are interviewed individually and in groups at each centre.

**Findings:**

The hypothesis was expanded to a program theory. The compliance of the professionals was the most significant element that explained why the open access service facilitated positive parenting. The professionals act in a compliant manner to meet the needs of the children and parents as well as in creating good conditions for social networking and learning amongst the parents.

**Conclusion:**

The compliance of the professionals in this program theory of open pre-schools at family centres can be a standard in integrated and open access services, whereas the organisation form can vary. The best way of increasing the number of integrative services is to support and encourage professionals that prefer to work in a compliant manner.

## Introduction

The practice of family centres is built on the knowledge that parents are often insecure in the new situation of being a parent with a new-born child [1]. The family centre movement can most of all be seen as a response to parents' needs that appear mostly during the first years of parenthood. Their abilities vary and are dependent on the psychological and social resources that are available in or around the family [2]. Parents’ levels of stress and need of support also vary within and between families from day to day, and are difficult to predict [3]. The integrative family centre program therefore provides protective factors to meet parents’ various abilities and needs in a dynamic way [2].

Family centre services are found in France, Greece, New Zealand, UK, Canada, Germany, Ireland and USA [4]. These services can be organised in various ways with different professions. In Finland, Norway, and Sweden, the family centres are usually co-located facilities that run family support programmes in an inter-professional context [5].

The family centre is a complement to the general welfare program in Sweden. Thus, it has a universal objective that promotes health to all families living within a geographical area. In Sweden, the Swedish maternal and postnatal child health cares at the family centres are important parts of the interdisciplinary service [6]. Family centres in Sweden run by the public sector have branched out during the last two decades, and the numbers of centres have increased continuously up to date. The professionals working at these integrated health care service centres are employed by and professionally linked to, their parent organisations of e.g., maternal care, health care, social services and open pre-schools, respectively [7, 8]. In these settings, district nurses, midwives, social workers and pre-school teachers work parallel to each other with the same target group—that is prospective parents and families with children below the age of six. However, parents with new-born children or toddlers are the most frequent visitors. Originally, the service aimed to target those families that are at risk of falling between the cracks of different specialized welfare services [8].

Central to the family centre activities in Sweden is the drop-in service which is called the *open pre-school*, where parents visit the open pre-school *together* with their children [8]. The service is accessible to parents while they are on parental leave or have leisure time on week days. This is a unique component in comparison to family centres in other countries such as Great Britain, since the purpose of the services in Sweden is not to provide day care in order to enable parents to work [8]. The open pre-school has in earlier research been seen as providing “... self-realisation in which parents have the possibility to use both expert and lay knowledge in improving their lives and the lives of their children” [8, p. 143]. Parents can, *together* with their children, easily access family support at the open pre-school. The support is delivered mostly by pre-school teachers and social workers, but also on occasion by nurses and midwives [6]. The drop-in service is available if and when parents need support without a time appointment in advance.

In an evaluative research review of family centres in Sweden, the Swedish Board of Social Welfare [5] has described the strength of the program. Parents are satisfied having a multidisciplinary team working under the same roof. They also improved their social network and the children got new acquaintances. Further, parents felt recognized, appreciated and safer after they had visited the family centre [5]. However, the open pre-school program at family centres has not yet been explored in-depth. The aim of this study is to understand the successful outcomes by exploring the input of personnel and the working mechanism of the program.

## Methodology

The dynamic nature of a program must be considered when selecting an approach to explore and describe the implicit theory underlying program actions [9, 10]. According to Weiss, the theory in the open pre-school program at family centres consists of “experience, practice knowledge, and intuition, and practitioners go about their work without articulating the conceptual foundation of what they do” [10, p. 503]. The design of the study is focused on making the theoretical program mechanisms explicit but also to contribute to future theory-based evaluation of such programs [10, 11].

A multi-case design was used [12]. Six open pre-schools at family centres were chosen as cases. As Yin suggests, a hypothesis, based on earlier descriptive evaluation data from Sweden and other countries, was formulated [5, 6, 8, 13]. The hypothesis was used as a starting point in order to explore similarities of and variation between the six family centres chosen (see square). The exploration gives the opportunity to continuously expand the hypothesis based on empirical findings.

**Square: Initial hypothesis**

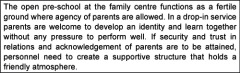

### Sample

Six family centres in the Western region in Sweden were selected purposively due to their richness of information on the program of open pre-schools. The inclusion criteria were the presence of experienced professionals, pre-school teachers and social workers, who work regularly at each setting. The selection also aims to uncover any variation among the open pre-schools depending on different types of housing such as urban (inner-city) or rural areas (towns in the countryside). This was done in order to explore if services vary in areas of different socio-economic dispositions. In Table 1, the characteristics of the family centres are described.

### Data collection

The analytical units were personnel and parents lived experiences of the open pre-school activity [14]. The procedure of the data collection and analysis was performed simultaneously in line with the logic of replication [12]. The researchers formulated a focus to explore at each site based on earlier knowledge of the professionals’ expertise and the characteristics of the area of the family centre (see Table 2).

The data collection consists of three steps. *The first step* was performed by one of the authors making telephone interviews with all the pre-school teachers and social workers working regularly at the open pre-school. At each site, two to four personnel were interviewed; 16 persons in total. The interviews were performed in dialogue form and consisted of open-ended questions focusing on work procedures and in what way they thought this would bring on change for the families, and why. Questions were: What do you want to achieve? What brings success and what is less successful to support parents’ and children’s development?

The *second step* consists of individual interviews with parents and was performed by both authors. At each site four to eight parents, 40 in total, were interviewed. All parents visited the open pre-school at the family centre on a more or less regular basis. The overall selection of the parents at the six family centres was made in order to mirror the variations of the parents in general in Sweden. The five selection criteria were; age, marital status, education, gender, and ethnicity. The interviews were performed in dialogue with open-ended questions that focused on how the parents experienced change for themselves as parents and for their children, due to their attendance in the open pre-school activity. Questions were: Why do you visit the open pre-school? What do you and your child gain from the visits? What is special about the open pre-school? Why does it matter to go there? How do you feel after you have been there?

*The third step* involves a group interview including a reflection dialogue. This was performed by the two authors with both parents and personnel attending. All parents already interviewed were invited. Two to five parents at each site attended the group interview together with all personnel, also interviewed previously. The authors summarised data during the session from these interviews and informants reflected on these summaries in a final reflection dialogue. The group session was performed in order to test the relevance of the analysis and to further increase the understanding of the program by reflections on the preliminary analysis. Main findings from the data collection at each family centre including the group interviews are shown in Table 2.

### Analysis of data

The interviews were transcribed verbatim, and the data were analysed preliminary after the data collection was finished at each family centre site (see above). The hypothesis was questioned deductively after each family centre, and the questions that arise in the findings from the group interviews at each family centre were further explored at the next site (see Table 2).

Data from the interviews with the parents, and the personnel, respectively were inductively analysed in the final analysis when all visits to family centres were finished. The interviews were read through by one of the authors to get an overview. Twenty-eight codes were identified and then analysed into eight categories using Atlas-ti version 5.5. The second author checked the relevance of the categories by reading the interviews. These categories formed the program theory (see Table 3).

### The final dialogue circle with personnel

The 28 codes and eight categories found in analysing the data from all six settings were further investigated in a dialogue session, in which 12 of the 16 personnel that previously had been interviewed attended. A dialogue circle technique was used as a way to stimulate creativity and acquire deeper awareness of the program theory [15]. Reflection and listening to others was encouraged by letting everybody have their say, one at a time. As the starting point for the dialogue, the question “What did the personnel do that was so good?” was raised.

During the dialogue circle, a so-called ‘creative loop’ took place [16] when the personnel jointly and suddenly realised that they had developed a dynamic way to relate to parents and children independently and on every site. The participants themselves chose to name the approach—professional compliance.

## The finding—the program theory of open pre-school at family centres

The hypothesis was expanded continuously when analysing data according to the eight categories, forming the program theory. The categories formed input and outcomes in the theory of the open pre-school program at the six family centres (see Table 3). The mechanisms were derived from relevant research related to data in the study, contributing to explanations of why the program worked.

The core finding of the program theory was the compliance of personnel to parents’ needs and abilities. The input theme came to be termed ‘professional compliance’ meaning personnel adapted according to parents’ situation and readiness for support. Professional compliance was thus the most significant element in why pre-school activity on the studied family centres rendered the outcomes.

In the following, the categories and the mechanisms are described.

### The input—how the program was performed

The objective of the professional’s input was to facilitate for parents to get psychological and social support earlier than they would have had in the ordinary specialized health and social care. The professionals complied with parents when they expressed needs and/or felt mature enough to receive the support.

“By emphasizing everyday problems, which were specific to individual parents, and making them generic and universal, the parents’ identity was strengthened. They get a chance to identify with other parents.” (pre-school teacher IV)

The personnel created space for the parents to reflect on their own experiences through dialogue. This dialogue took precedence over planned activities in daily practice. They encouraged parents to build their capacity as parents individually or in groups of parents. Parents of the shy and aloof kind acquired support through merely overhearing other more forthcoming and outspoken parents talking about their experience.

“I try to create dialogues of good quality where they can give voice to their thoughts on which they can reflect. I support them in how to find their answers and speak them out. In this way they will grow in their parenthood from within.” (pre-school teacher II)

In this way the parents were encouraged to develop their parenting in various ways. The professionals encouraged attachment bonds by emphasizing parents’ own resources in how to find solutions that were appropriate for the individual parent and based on their own specific child’s/children’s needs. This was done in order to ‘warm up’ the relation between parent and child—to encourage attachments bonds.

“We focus a lot on bonding, which is a lot about acknowledging. Many parents are hesitant in terms of having faith in themselves as to being able to soothe their child and feel mutual love. I talk about the positive characteristics that I see in the parents. I talk about how lovely their child is.” (pre-school teacher III)

The professionals acknowledged parents with all their positive and negative personal traits. Meanwhile the professionals were over-looking the parent-child relation. The message was that parents were accepted as they were, according to the ‘good enough’ parent. However, sometimes parents were encouraged to find different ways to act in the relation to the child.

### The outcomes in the program

From the parents’ view, the input from personnel as well as other parents and children in the context of the open pre-school were crucial to the rendered outcomes. The feeling of being a parent with a child just like any other parent with their children overrode the feelings of insecurity and being the only parent with problems. The setting implied safety and security along with feelings of warmth in which parents could relax and cast off the social facade of being ‘the skilled parent’. Hence, encounters and dialogues with other parents as well as the personnel at the open pre-school resulted in increased self-esteem and self-confidence. The parents felt empowered.

“You come here and feel proud. If you’ve had a rough time, you feel proud as a peacock when you hear someone say that your children are good. You flaunt your feathers a little more here, and when I come home I feel so strong.” (parent VI)

The parents were enabled to easier cope with problems of parenting. When a parent feels at ease, he/she can easier reflect on, and get a distance to, himself/herself. For example, in times of overwhelming nightly interruptions, the open pre-school offered relief for the parents.

“It helps a lot to leave all ‘have-to-dos’ at home to only ‘being’ here. Just being here, to see other people and just be as you are, makes you able to give more to your child. If I only stay home I will give some, but I want to give more than that.” (parent II)

Social networking was central to both parents and children, whether it had to do with making friends for life or making mere acquaintances. Children who were shy were able to approach other children or other grown-ups than their parents, at their own pace. Meanwhile, as the social networks of the parents grew, they also got access to the social support of one another.

### Mechanisms that explain the outcomes

The following mechanisms explained the outcomes and were identified through the use of relevant theories in literature. The theories supported explanations to why the theme of the study—professional compliance—rendered the outcomes. The following mechanisms were identified: the psychological, the educational/pedagogical and the social.

*The psychological mechanism* covered the opportunity to form an identity as a parent and encouraged attachments bonds between parent and child [17]. The approach—professional compliance—empowered and encouraged parents to act as auxiliary resources for one another in the creation of a parent identity. Obviously, to create a dynamic and supportive environment inhibiting a welcoming atmosphere that facilitated opportunities for a positive parent identity was important in professional compliance. In the following quote, a parent gave voice to an example of this, after having developed an awareness of and a more realistic view on what a ‘good enough’ parent might entail:

“For me the personnel have meant a lot since I am a single mother. I have received great support when in doubt of being a good enough parent. I was not able to appreciate the happiness and the positive things about my child; instead I was preoccupied with the negative things.” (parent I)

The fertile ground for the *educational/pedagogical* mechanism came from the novelty of becoming a parent. Parents were strongly motivated to give their children a good start in life [18]. The professional compliance encouraged parents’ own resources in a sensitive manner to meet their learning needs and readiness, and facilitated parents’ feelings of empowerment. The learning environment was influenced by theories of empowerment and liberation of individuals [19], learning by doing [20] and social learning theories [21]. In short, everybody learned and everybody taught. This learning ‘smorgasbord’ consisted of shared experiences and mutual learning opportunities [22].

The *social mechanism* covered the availability of the family centre as an arena in which parents built social networks that could facilitate the coping with stress [2, 3]. The professional compliance approach facilitated the creation of an informal social network that might remind us of a persistent family [2, 13]. Parents interacted actively in dialogues or merely listened to others while they played with their child/children. They chose whether to be close or not, and this created the space and opportunity for bringing more private problems to the fore.

“It is so important that parents can relate to other parents, and that they are allowed to just be here without feeling required to expose themselves. They may be talking to other parents and very next minute, they may be sitting in the corner talking to me, it becomes natural after a while. They do not have to make a deal about it.” (social worker)

The social network was attained because of the access of other parents with whom they could choose to share their experiences in how to be a parent today and in the future.

The professional compliance to parents’ relative readiness to receive psychological and social support and to learn was the driving force to why the program rendered the outcomes.

## Discussion

The hypothesis was expanded on the program theory. Professional compliance to the readiness of the parents was found to be the most significant element explaining why the input in the context of the open pre-school activity rendered the outcomes. This finding corresponds to Warren-Adamsson and Lightborn [2] who claims that sensitivity in the relationships to parents would be recognised as the core of family centre practice. Moreover, in a literature review, the family centre-based practice was seen as a containing space for parents to mature [13]. In line with the findings of Nyström and Öhrling’s [23] literature review, professional compliance meets parents’ anxiety in becoming parents living in a new and overwhelming world with multiple changes. A comprehensive open pre-school service could thereby function as a ‘smorgasbord’ for parents from which they could choose what they believed might be working for them, whenever they might need it [22].

However, open access services in general can be organised differently than family centres in the Nordic countries. In Australia, an appointment-free and parent-led service was organised in a child health surveillance clinic. Both personnel and parents found the service to be effective, parent-directed and flexible in contrast to individual appointments [24]. Barnes et al. [25] focused on first time mothers in Australia, when finding that dynamic open access services is an answer to meet parent empowerment needs. So, the standard that is common to most open access services is the professional compliance component, whereas the organisational form can vary.

Similarly, the professional compliance component can also be relevant when it comes to other target groups. For instance, success in a drop-in program focusing on youth was found to be dependent on the dynamic, openness, availability and understanding of the personnel’s qualities as a core component [9]. Professional compliance could thus be an indicator of good quality in meeting the instant and fluctuating needs in various target groups.

However, this kind of dynamic service places high demands on the personnel. They need to cope with input work that is often loosely structured and in line with Moxnes [26], unstructured activities may create organisational anxiety that might challenge professionals. Usually, professional actions are formed on a set of rules, role descriptions, limitations and responsibilities [27] that establish a structure as protection against anxiety [26]. However, following Moxnes [26] professional compliance can provide dimensions for a social structure; interpersonal relationships and ways of thinking and working. An awareness and internalisation of a professional compliance in everyday work can, in this way, provide a structure that balances organisational anxiety among professionals. If they are unable to cope with this kind of structure, they will probably have difficulties in contributing to the supportive mechanisms which explain the outcomes in these kinds of dynamic services.

How does this social structure develop? Does this kind of dynamic and integrative service support individual professionals who feel fettered in their professions and give them opportunity to push for new initiatives? Originally the initiative for the establishment of family centres in Sweden was derived from ‘front-line workers’ among different professions. The increase of family centres in Sweden has rather been a bottom-up social movement than an initiative of legislation [28]. Thus, sensitive and responsive individuals among the personnel who have chosen to work in an integrative service are intertwined with the increase of family centres. So, the Swedish example suggests that the best way of increasing the number of integrative and dynamic services is to support and encourage professionals that prefer to work in a compliant manner.

Another question that arises is how professional compliance is facilitated in an integrative service with different professions in order to create the best possible conditions? The traditions based on different norms and values between individuals in health and social professions can respectively hinder a positive development of the family center service [7]. The need to combine different professional skills in order to realize the potential of an integrative service was highlighted by O’Brian [29]. He found that if the difficulties in an inter-professional context were not addressed, the outcome of the service would be negative for the parents and their children. The conclusions in both studies [7, 29] were that the professional commitment to the service user must be complemented with measures to improve inter-professional communication.

The collaborative and iterative process in exploring the program theory provided opportunities for the personnel to flourish from encouragement and to reflect on how they carried out their work and why, which implied work-based learning [11, 30–32]. This became obvious during the final dialogue session when the collective of personnel learned through a creative loop stemming from their independent experiences from each site. The creative loop is an example of how a highly interactive and creative approach [16] can contribute to make experiences explicit and to further develop the understanding of a program theory [11]. The creative learning process has contributed with knowledge used in the final development of the initial hypothesis to a comprehensive program theory.

The weakness of this study is that despite family centres being an integrative health and social service, the professions of nurses and midwifes were not included in the study. They could have raised the issue of health content quality significantly [29], and might have contributed to an even more comprehensive program theory.

The complex design of the study was planned in order to increase external validity [12]. The strength is the variation between the six different sites included in the development of this program theory [11]. The program theory in question is built on triangulation of different samples and methods of data collection. Both the personnel and parents participated in an interactive and iterative process. Despite the variation between the sites, the commonality—professional compliance—is the central finding. If the personnel recognise their service in this program theory, it can be transferred primarily to other open access services in family centres, but also to open access services in other organisational forms.

Even though a program theory strives to illustrate the process as being linear, it could be seen as a schematic description of input and outcomes as well as the mechanisms that explain the outcomes in open-access services. The professional compliance with parents’ instant needs is a soft, sensitive and complex issue situated in a context and therefore it is difficult to investigate fully [33]. All of these non-linear and complex implications should be taken into consideration when evaluating a program theory of this kind.

## Conclusion

The compliance of the professionals in this program theory of open pre-schools at family centres can be a standard in an integrated and open access services, whereas the organisation form can vary. The best way of increasing the number of integrative services is to support and encourage professionals that prefer to work in a compliant manner, and complement this with measures to improve inter-professional communication. A future evaluation can use this program theory but needs to acknowledge the non-linear and complex process in the relationships between professional compliance and parents' needs.

## Reviewers

**Vincent Busch**, PhD Student, Julius Center for Health Sciences and Primary Care, University Medical Center Utrecht, The Netherlands

**Karin Forslund Frykedal**, Fil Dr., Senior Lector, Department of Behavioural Sciences and Learning, Linkoping University, Sweden

**Debbie Watson**, Dr., Senior Lecturer Childhood Studies, School for Policy Studies, University of Bristol, UK

## Figures and Tables

**Table 1.**
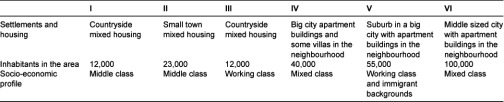
Characteristics of each of the six family centres.

**Table 2.**
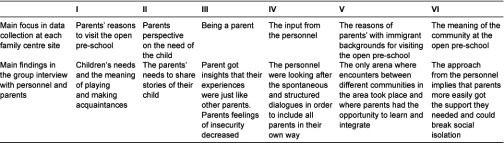
The logic of replication—main focus at each family centre of the data collection, and the main findings in the group interview with personnel and parents.

**Table 3.**
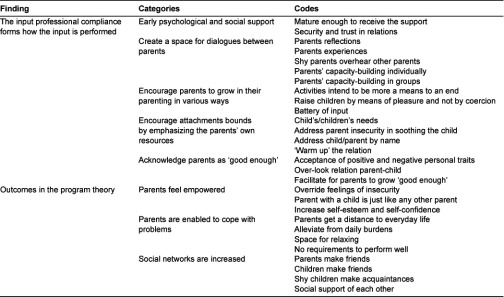
The codes and categories of the input and outcomes in the program theory.
